# Treatment of Persons Apparently Drowned

**Published:** 1857-07

**Authors:** 


### TREATMENT OF PERSONS APPARENTLY DROWNED.

THE Secretary of the Royal National Life Boat Association requests us to publish a series of rules, for the treatment of persons apparently drowned. We give with pleasure the really useful points.

ROYAL HUMANE SOCIETY'S INSTRUCTIONS. Send quickly for medical assistance. *Cautions.*—1. Lose no time; 2. Avoid all rough usage; 3. Never hold up the body by the feet; 4. Nor roll the body on casks; 5. Nor rub the body with salt or spirits; 6. Nor inject tobacco-smoke or infusion of tobacco. I. Convey the body carefully, on its face, with the head and shoulders supported in a raised position, to the nearest house. II. Strip the body and rub it dry; then wrap it in hot blankets and place it in a warm bed in a warm chamber, free from smoke. III. Wipe and cleanse the mouth and nostrils. IV. In order to restore the natural heat of the body, move a heated covered warming-pan over the back and spine; put bladders or bottles of hot-water, or heated bricks, to the pit of the stomach, the arm-pits, between the thighs, and to the soles of the feet; foment the body with hot flannels; and rub the body briskly with the hand; do not, however, suspend the use of the other means at the same time; but, if possible, immerse the body in a warm bath at blood heat. V. Volatile salts are to be passed occasionally to and fro under the nostrils. VI. No more persons are to be admitted into the room than are absolutely necessary. If apparently dead from intense cold, rub the body with snow, ice, or cold water; restore warmth by slow degrees; it is highly dangerous to apply heat too early.—*General Observations.* On the restoration of life, give small quantities of wine, or diluted brandy, warm, keeping the patient quiet in bed. The treatment is to be persevered in for three or four hours. It is absurd to suppose that a body must not be meddled with or removed without the permission of a coroner.

Dr. Marshall Hall's Instructions. Treat the patient instantly, on the spot, in the open air, expose the face and chest to the breeze (except in severe weather). I. *To clear the throat,* place the patient gently on the face, with one wrist under the forehead. If there be breathing, wait; if not, or if it fail, II. *To excite respiration,* turn the patient well and instantly on his side, and excite the nostrils with snuff, or the throat with a feather, etc., and dash cold water on the face previously rubbed warm. If there be no success, lose not a moment, but instantly, III. *To imitate respiration,* replace the patient on his face, raising and supporting the chest well on a folded coat or other article of dress; turn the body very gently on the side and a little beyond, and then briskly on the face, alternately; repeating these measures deliberately, efficiently, and perseveringly, fifteen times in the minute, occasionally varying the side; when the patient reposes on the chest, this cavity is compressed by the weight of the body, and expiration takes place; when he is turned on the side, this pressure is removed, and inspiration occurs. When the prone position is resumed, make equable but efficient pressure, with brisk movement, along the back of the chest; removing it immediately before rotation on the side. IV. *To induce circulation and warmth* rub the limbs upwards, with firm grasping pressure and with energy: by this measure the blood is propelled along the veins towards the heart. Avoid the continuous warm-bath, and the position on, or inclined to, the back.

